# *SAG3 Toxoplasma gondii* cloning reveals unexpected fivefold infection in the blood of feral cats in the Mexican Caribbean

**DOI:** 10.1186/s12917-021-03129-9

**Published:** 2022-01-14

**Authors:** Luis Fernando Valenzuela-Moreno, Sara Teresa Méndez-Cruz, Claudia Patricia Rico-Torres, Carlos Cedillo-Peláez, Dolores Correa, Heriberto Caballero-Ortega

**Affiliations:** 1 Laboratorio de Inmunología Experimental, Instituto Nacional de Pediatría, Insurgentes Sur 3700-C, Colonia Insurgentes-Cuicuilco, Delegación Coyoacán, Insurgentes Sur 3700C, Col. Insurgentes Cuicuilco, C.P. 04530 Ciudad de México, México; 2 Laboratorio de Bioquímica Genética, Instituto Nacional de Pediatría, Insurgentes Sur 3700-C, Colonia Insurgentes-Cuicuilco, Delegación Coyoacán, C.P. 04530 Ciudad de México, México

**Keywords:** *Toxoplasma gondii*, Genotyping, Feral cats, Mixed infections, Mexican Southeast, Cloning

## Abstract

**Background:**

Currently, more than 300 genotypes of *Toxoplasma gondii* (*T. gondii)* have been described throughout the world, demonstrating its wide genetic diversity. The *SAG3* locus is one of the genes included in the genotyping panel of this parasite. It is associated with its virulence since it participates during the invasion process of the host cells. Therefore, cloning, sequencing, and bioinformatic analysis were used to deepen the understanding of the *SAG3* locus genetic diversity of *T. gondii* in blood samples from feral cats.

**Results:**

Six different *SAG3* sequences were detected, five of which were detected in one feline. Three sequences were first reported here; one of them was an intragenic recombinant. In the cladogram, four out of ten *SAG3* sequences did not share nodes with others reported worldwide.

**Conclusions:**

Cloning and sequencing of samples with more than one restriction pattern by PCR-RFLP were very helpful tools to demonstrate the presence of more than three genotypes of *T. gondii* in the blood of feral cats from southeastern Mexico. This suggests a potential mixed infection of multiple *T. gondii* strains and high genetic diversity of the parasites in felines in this tropical region of Mexico.

**Supplementary Information:**

The online version contains supplementary material available at 10.1186/s12917-021-03129-9.

## Background

*Toxoplasma gondii* is one of the most successful parasites in the world because of its ability to infect and persist in most warm-blooded animals [1]. The presence of this parasite has been demonstrated in felines on all continents, including Antarctica [2, 3]. Currently, the only definitive hosts for *T. gondii* are members of the Felidae family (wild and domestic) since sexual reproduction of this parasite takes place within their small intestine. If a feline ingests tissue cysts or oocysts of different genotypes simultaneously, recombinant strains can be produced. Intragenic recombinant genotypes can be generated during the sexual reproduction of *T. gondii*. In addition, during the asexual reproduction cycle within intermediate hosts (and in nonintestinal epithelial cells of felines), single nucleotide polymorphisms (SNPs) may arise [4, 5]. Regions with a tropical climate and a high density of definitive hosts are expected to have a high genetic diversity of the parasite and a lower probability of finding clonal genotypes/sequences [6].

Previously, we cloned and obtained sequences that are identical to known highly virulent strains as well as to new ones [7, 8]. *SAG3* is a locus involved in the parasite invasion process, and it is associated with its virulence [9, 10]. We have been successful in amplifying and genotyping this marker by PCR-RFLP from clinical samples, detecting several mixed infections [7, 8, 11, 12]. We previously reported two triple infections (I + II + III) by RFLP in blood samples of feral cats from the Mexican Caribbean, a finding that is becoming quite common in the country. Thus, we performed a more profound approach and evaluated the degree of genetic diversity of *SAG3* at the sequence level; we also compared the results to those published in genetic databases and we present the results herein.

## Results

**Fig. 1:**
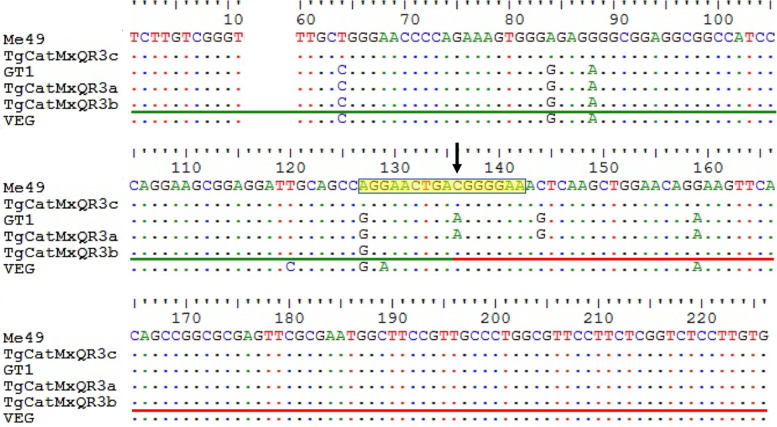
Multiple sequence alignment of the *SAG3* locus of *Toxoplasma gondii*. Blood samples were obtained from a cat (TgCatMxQR3) from Playa del Carmen, QR. The three cloned sequences from the sample (**a**, **b** and **c**) were aligned with *SAG3* sequences of reference GT1, Me49 and VEG strains (types I, II and III, respectively). In the TgCatMxQR3b sequence, a recombinant intragenic sequence was determined (arrow): identical regions of GT1 (type I) and Me49 (type II) strains were found and are highlighted in green and red underlines, respectively. The recombination predicted site is highlighted in the yellow box. The alignment was performed with BioEdit v5.0.6 software and sequences with Gene IDs: GT1, TGGT1_308020; Me49, TGME49_308020; VEG, TGVEG_308020 in www.toxodb.org

Ten of the eleven feral cats sampled were positive for infection by *T. gondii,* and two of these ten cats (TgCatMxQR3 and TgCatMxQR6) had mixed infections detected by PCR-RFLP at the *SAG3* locus*,* which was cloned and sequenced. From the PCR product of TgCatMxQR3, we obtained 15 positive colonies that contained alleles I and II (no colony had a type III allele). From these, we randomly selected four clones, two type I and two type II alleles. Among these clones, three were identical to the reference strains: TgCatMxQR3a (one clone) was identical to the GT1 strain (type I), while TgCatMxQR3c (two clones) was identical to Me49 (type II). Clone TgCatMxQR3b was an intragenic recombinant I × II, since the sequence from nucleotide 1 to 135 was identical to GT1 (type I), and from 136 to 226 it was identical to Me49 (type II) (Fig. 1).

**Fig. 2:**
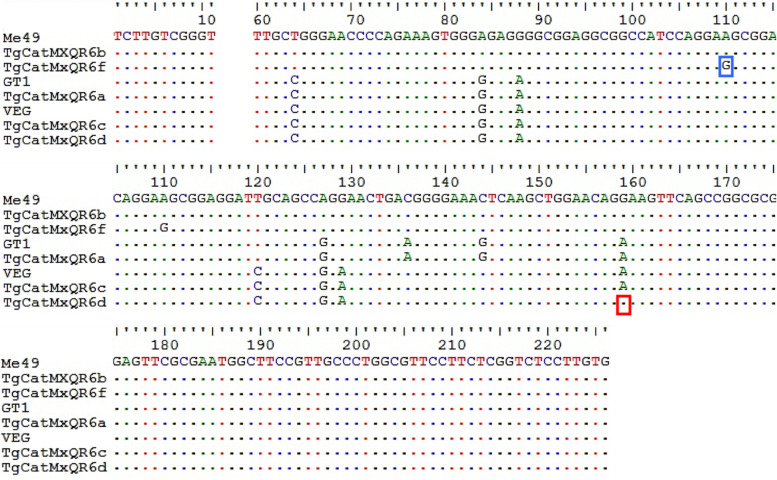
Multiple sequence alignment of the *SAG3* locus of *Toxoplasma gondii*. Blood samples were obtained from a cat (TgCatMxQR6) from Playa del Carmen, QR. The five cloned sequences from this cat (**a**, **b**, **c**, **d**, and **f**) were aligned with *SAG3* of GT1, Me49 and VEG reference strains (types I, II and III, respectively); the sequence of TgCatMxQR6e was ruled out due to spurious SNPs. Polymorphisms shared between Chinese and Mexican strains are highlighted in the blue box, and type II polymorphisms are highlighted in the red box. The alignment was performed with BioEdit v5.0.6 software and sequences with Gene IDs: GT1, TGGT1_308020; Me49, TGME49_308020; VEG, TGVEG_308020 in www.toxodb.org

Additionally, we obtained 15 *SAG3*-positive colonies from the PCR product of TgCatMxQR6 that had type I, II and III alleles. From these, we selected six colonies to purify the plasmid and subsequently sequenced them (one type I, three type II and two type III). The TgCatMxQR6a, TgCatMxQR6b and TgCatMxQR6c sequences were identical to the reference strains GT1, Me49 and VEG (type I, II and III), respectively. The TgCatMxQR6f sequence was almost identical to Me49 but had a single nucleotide polymorphism (SNP) at the 110 (A/G) position. Finally, TgCatMxQR6d has a SNP at position 159, which makes it different from the VEG strain (Fig. 2). In summary, six different *SAG3* sequences were detected, five of which were detected in feline TgCatMxQR6.

All SNPs found in the sequences obtained from TgCatMxQR3 and TgCatMxQR6 were verified in the sense and antisense chromatograms from both sequencing institutions. For the 226 bp region of the *SAG3* gene, we analysed 196 sequences, and 14 different haplotypes were identified, which contained 15 polymorphic sites. We predicted four recombination regions: 62–86, 86–108, 108–118 and 127–142 bp. The recombinant site of the TgCatMxQR3b sequence was located in the 127–142 bp region (Fig. 1).

**Fig. 3:**
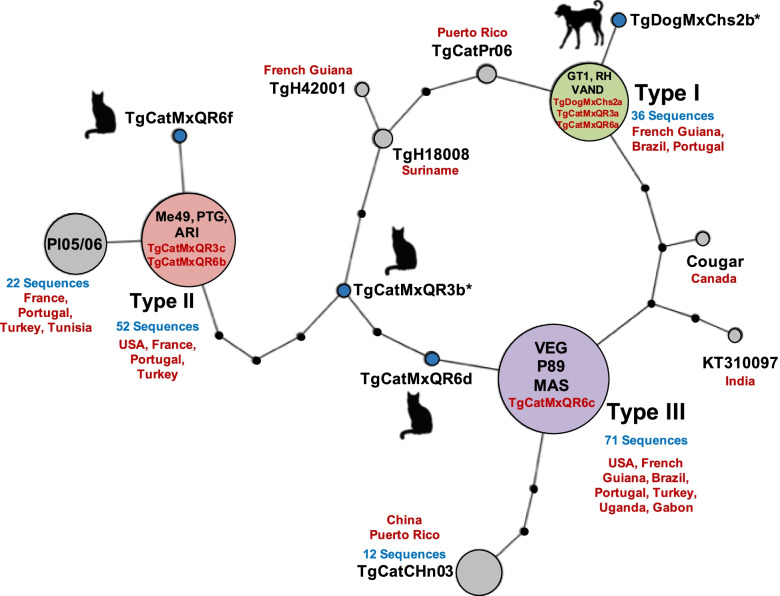
Haplotype network based on *SAG3* sequences of *Toxoplasma gondii*. The network was built by a statistical parsimony method. The size of the circles is proportional to the number of sequences. Black nodes indicate unsampled or extinct haplotypes; blue nodes represent Mexican haplotypes that were not grouped into sequences previously reported. Asterisks (*) denote recombinant sequences. TgDogMxChs2a and TgDogMxChs2b were isolated and previously reported [8]. A cladogram was generated with TCS Beautifier using the TCS algorithm (https://cibio.up.pt/software/tcsBU/)

With the alignment generated, a haplotype network was built using the TCS algorithm (Fig. 3). The classical allele type III grouped the majority of sequences aligned (71), followed by type II (52) and type I (36). Six out of ten sequences obtained by us, including from a dog in Chiapas (TgDogMxChs2a) that was previously reported [8] were grouped into the nodes of reference strains I, II and III: TgDogMxChs2a, TgCatMxQR3a, and TgCatMxQR6a (type I); TgCatMxQR3c and TgCatMxQR6b (type II); and TgCatMxQR6c (type III). The remaining four sequences, including TgDogMxChs2b, did not share nodes with any other aligned sequence. The eight sequences obtained from cats (three with unique polymorphisms and five identical to the type I, II or III reference strains) were deposited in GenBank with accession numbers MW281504, MN562751, MW281505, MW281506, MW281507, MW281508, MN562752, and MN562755.

## Discussion

The acquisition of nucleotide sequence data of *Toxoplasma gondii* is hard to achieve due to the difficulty of isolating the parasite from asymptomatic hosts; thus, researchers must attempt genotyping from clinical samples where the DNA of the parasite is heavily diluted within the host’s DNA [5, 13]. To deepen the analysis of the mixed infections found in the *SAG3* locus, we cloned and sequenced DNA from clinical samples that showed a triple infection pattern by PCR-RFLP.

Mixed infections due to *T. gondii* are demonstrated by PCR-RFLP when more than one restriction pattern is visualized and by sequencing when two peaks overlap due to different nucleotides at the same position [11, 14, 15]. However, one disadvantage of PCR-RFLP is that a maximum of three coinfections can be suspected when the restriction patterns of the three classic alleles are visualized for a given locus, just as we reported for the *SAG3* marker in clinical samples from feral cats in Quintana Roo [7].

By cloning the PCR amplification products, we confirmed the mixed infections of TgCatMxQR6 and TgCatMxQR3, demonstrating the presence of five and three different sequences, respectively. From the isolated clones, we obtained classical alleles (I, II and III) as well as others that harboured unique SNPs. This is an unprecedented result, since, as far as we know, there is no prior evidence of more than three different *T. gondii* genotypes in a single blood sample [14]. The result is so surprising that we thought it might be due to cross contamination in the laboratory, mistakes introduced by the polymerase or chimaeras produced during the PCR procedures. However, during the amplification and cloning assays, we included appropriate controls to assure there was no contamination, and in every PCR assay, hot-start high-fidelity AmpliTaq Gold™ polymerase was used, which has a barely noticeable error rate (2.6 × 10^5^) [16]. To reduce the probability of chimaera production, we also implemented several technical measures, such as an increased elongation time (1.5–2.0 min per cycle), low quantity of DNA as a template (ratio of the host:parasite DNA 1 × 10^−5^), a reconditioning step (dilution 1:2 of the amplification products of the multiplex PCR before the nested PCR) and the use of DMSO as a reaction stabilizer [17–19]. In addition, DNA from only one of the reference strains was used, and most of the sequences differed from the control strain (RH).

This phenomenon has also been reported in Malawi and Cambodia in patients infected with the related apicomplexan *Plasmodium*, where the use of nPCR allowed the identification of up to four genetic variants, but when they applied high-resolution molecular techniques (massively parallel pyrosequencing -MPP-), they were able to identify up to 16 genetic variants in samples that had previously yielded three genotypes only [20]. These results have implications for the prognosis of the disease and drug therapy because it has been reported that when patients are infected with several strains of *Plasmodium*, drug treatment can “help” some variants to perform better after removal of other competitor strains [21]. This could also occur in mixed *T. gondii* infections in hyperendemic regions, where some strains may be resistant to some chemotherapeutics and are the cases that do not respond to the current treatment against the parasite. In addition, the two sampled cats were feral, and these animals obtained their whole diet from hunting small mammals and birds; if the cats consume prey infected with different genotypes within several days, it is possible that more than three genotypes could be found circulating in their blood because tachyzoites can be found up to 10 days after oral infection [22–24].

Two unique polymorphisms were found in the sequences TgCatMxQR6d and TgCatMxQR6f. The first of them (A/G, 159 position) is a type II strain SNP, while the second (A/G, 110 position) has been reported in sequences isolated from China (GenBank® KU599378, KU599384, KU599385, and KU599386); however, both sequences are new. The finding of sequences that carry unique SNPs in Mexico has also been reported in pigs from Yucatan: Cubas-Atienzar et al. [15] found 18 different Alt. *SAG2* sequences in addition to the I and III classic alleles in blood and muscle samples of 40 swine. One of the three sequences found here (TgCatMxQR3b) that did not have 100% identity with those reported elsewhere is an intragenic recombinant between type I × II. This phenomenon has also been reported in one dog from Chiapas, where a recombinant sequence I × II was reported and it produced an atypical *SAG3* allele [8]. Cubas-Atienzar et al. [15] also reported I × II intragenic recombinants at Alt. *SAG2* and *SAG3* markers and one I × II × III at the *GRA6 locus*. Therefore, these results suggest that there are more intragenic recombination events than previously thought, and they are frequent in hyperendemic regions.

Four out of ten sequences of *SAG3* reported here, including one from a dog from Chiapas, were located in unique nodes of the cladogram, in some cases up to five mutational steps far away from the classic I, II, and III alleles, which confirms our hypothesis that there are endemic *T. gondii* strains in Mexico. The other six sequences were grouped inside the nodes of the classic alleles, with sequences obtained mainly from the USA and Europe (France, Portugal and Turkey), where genetic diversity of the parasite is reduced. This may be due to the presence of few native species of felids in these regions (other than feral domestic cats); in the USA and Canada, only cougar (*Puma concolor*) and red lynx (*Lynx rufus*) are naturally distributed; and in Europe, there are only three species: European wild cat (*Felis silvestris silvestris*), northern lynx (*Lynx lynx*) and Iberian lynx (*Lynx pardinus*), so there would be a lower probability of genetic recombination events [25]. In the smallest nodes, isolates from tropical regions of South America and the Caribbean were found, where there are up to nine species of felines besides the domestic cat, and therefore greater genetic diversity of the parasite [25].

Jiang et al. [26] analysed the frequency of genotypes isolated in the USA from domestic and wild animals and concluded that in domestic/urban transmission cycles, new genotypes are rarely found, while in the sylvatic transmission cycle, there is high genetic diversity (up to 10-fold) with a greater frequency of new and atypical strains. The cats included in the present study lived in a sylvatic environment where they coexisted with five wild feline species [27].

## Conclusions

Cloning and sequencing of samples with more than one restriction pattern by PCR-RFLP demonstrated the presence of more than three genotypes of *T. gondii* in individual blood samples obtained from feral cats in Quintana Roo. This reinforces the existence of endemic strains in Mexico, high genetic recombination of the parasite and frequent exposure by felines to *T. gondii* of diverse genotypes. Isolation of live parasites is still necessary to confirm mixed infection in a given animal host.

## Methods

### Ethical considerations

The present study followed national and international regulations for animal welfare and care. It was authorized by the Review Board of the Instituto Nacional de Pediatría of the Ministry of Health of México (INP; IRB-NIH numbers IRB00008064 and IRB00008065), which includes the Research and Animal Care Committees (approved protocols 2012/013 and 2020/039).

### Origin of the samples

The DNAs used in this study were obtained from the blood samples of eleven feral cats captured in the municipality of Solidaridad, Quintana Roo, México, as previously reported [7]. In accordance with the eco-archaeological park annual undesired fauna control program, the owners of this private collection granted permission to capture the feral cats used in this work. All cats were anaesthetized using 9.7 mg/kg of tiletamine/zolazepam (Zoletil 100®, Virbac, Carros, France). Six milliliters of whole blood from the jugular vein were collected prior to euthanasia with a 3 mL IV of sodium pentobarbital (Dolethal®, Vetoquinol, France), performed according to the Official Mexican Standard NOM-033-ZOO-1995. The DNA of two cats (TgCatMxQR3 and TgCatMxQR6) was selected for further analysis because triple infection (I + II + III) was previously demonstrated at the *SAG3* gene of the parasite.

### DNA extraction

Using a commercial kit and following the manufacturer’s instructions (Gentra Puregene Tissue I kit, Quiagen), DNA from the blood was extracted and quantified on a NanoDrop 1000™ spectrophotometer (Thermo Scientific, Ma, USA) and kept at −20 °C until use.

### PCRs and cloning

To obtain the *SAG3* amplicons, the multiplex PCR products were diluted 1:2 in sterile water (10 μL + 10 μL), and using the internal primers and PCR conditions described previously, a 226 bp product from the DNA of cats TgCatMxQR3 and TgCatMxQR6 was amplified [28]. Briefly, PCR was carried out in a volume of 50 μL with each internal forward and reverse primers *SAG3* InF: 5′-TCTTGTCGGGTGTTCACTCA-3′ *SAG3* InR: 5′-CACAAGGAGACCGAGAAGGA-3′), DMSO 5%, 2 U AmpliTaq Gold™ (Applied Biosystems) and 1.5 μL of the 1:2 dilution as a template. Amplification was performed for 35 cycles with an annealing temperature of 60 °C. The PCR products were resolved on 1.5% agarose gels stained with ethidium bromide and then photodocumented (Bio-DocIt, UVP™). Two amplicons were obtained from each DNA sample; one of them was cut out and purified from the agarose gel using a commercial kit following the manufacturer’s instructions (Zymoclean™ Gel DNA Recovery Kit, Zymo Research, Cat. D4002, USA). The purified product was treated with DNA blunting enzyme for cloning and subsequent sequencing. The second amplicon obtained was digested with 7 U of the *Nci*I enzyme (New England, Biolabs®, R0196S, USA) and incubated at 37 °C overnight as described by Su et al. [28] to corroborate the mixed infection suggested by the RFLP pattern. The purified *SAG3* products were cloned into a pJET1.2/blunt cloning vector using T4 DNA ligase, incubated at 22 °C for 2 h and used to transform competent *Escherichia coli* Top 10 F′ strain using a commercial kit (GeneJET® PCR Cloning Kit, Thermo Scientific, Cat. K1231, USA) following the methodology described by Valenzuela-Moreno et al. [7]. Briefly, the transformed bacteria were grown in Luria-Bertani (LB) agar with ampicillin (100 μg/mL) at 37 °C overnight. Individual colonies were selected and reseeded in LB culture medium at 37 °C overnight. Colony PCR was performed using the internal *SAG3* primers to select the positive colonies, and the product was subjected to enzymatic restriction to determine the allele type. Finally, the selected colonies were propagated in 7 mL of LB culture medium, and plasmids were extracted and purified from positive colonies using a commercial kit (GeneJET® Plasmid Miniprep Kit, Thermo Scientific, Cat. K0503, U.S.A.). Purified plasmids were sequenced at the Instituto de Biología-UNAM and the Instituto Nacional de Medicina Genómica, México, using the sense and antisense primers included in the commercial plasmid kit. Chromatograms were analysed with SnapGene viewer® v 4.1.4. The consensus sequence was obtained after comparing the sense and antisense sequences. The Phred average quality was over 30 in all sequences obtained. All PCR assays were carried out with AmpliTaq Gold™ (Thermo Fisher Scientific, Cat. 4311806, USA). As a positive control, DNA of the RH reference strain (type I) was included, and sterile water was used as a negative control. For the PCR-RFLP assays, DNA of the RH, Me49 and VEG reference strains were included as type I, II and III controls, respectively. Polymerase fidelity and all reagents used for the PCR-RFLP and cloning were validated and are in the process of being published (Rico-Torres et al. unpublished).

### Bioinformatic analysis

Sequences obtained in this study were aligned using BioEdit v5.0.6 software and compared with *SAG3* sequences from 17 *T. gondii* reference strains downloaded from ToxoDB® (GT1, Me49, VEG, ARI, CAST, Cougar, CtCo5, FOU, GAB2–2007-GAL-DOM2, MAS, p89, RUB, TgCatBr5, TgCatBr9, TgCatPRc2, TgCkUg2 and VAND; https://toxodb.org/toxo/app/search/organism/GenomeDataTypes/result), 179 downloaded from GenBank (https://www.ncbi.nlm.nih.gov/genbank/) and two previously obtained by us from blood samples of a dog from Chiapas state, México (GenBank accession: MK127861, MK127862) [8] using the ClustalW® algorithm (Additional file). To determine polymorphic sites and the number of haplotypes, we used the DNA Polymorphism tool. In addition, the number of recombination events was determined by the Recombination tool; both are available in DNAsp v5.10 software. Finally, we established the genetic relationships from the aligned sequences with TCS v 1.21 and tcsBU (https://cibio.up.pt/software/tcsBU/) using statistical parsimony to build a haplotype network [29, 30]. All cloned sequences were subjected to *in silico* digestion using the online Benchling tool to confirm the RFLP pattern (www.benchling.com).

## Supplementary Information

**Additional file 1.**

## Data Availability

Reference strain sequences GT1, TGGT1_308020; Me49, TGME49_308020; VEG, TGVEG_308020 are available at www.toxodb.org. Genomes of ARI, CAST, Cougar, CtCo5, FOU, GAB2–2007-GAL-DOM2, MAS, p89, RUB, TgCatBr5, TgCatBr9, TgCatPRc2, TgCkUg2 and VAND strains are available at https://toxodb.org/toxo/app/search/organism/GenomeDataTypes/result. TgDogMxChp2a MK127861 and TgDogMxChp2b MK127862 are available at https://www.ncbi.nlm.nih.gov/genbank/. The sequences from cats were submitted and deposited in GenBank with the accession numbers MW281504, MN562751, MW281505, MW281506, MW281507, MW281508, MN562752, and MN562755. The accession numbers of the 179 GenBank® sequences downloaded are available in the Additional file
